# Sexual Abuse of Older Residents in Nursing Homes: A Focus Group Interview of Nursing Home Staff

**DOI:** 10.1155/2015/716407

**Published:** 2015-05-11

**Authors:** Maria Helen Iversen, Astrid Kilvik, Wenche Malmedal

**Affiliations:** ^1^Faculty of Nursing, Diakonhjemmet University College, Oslo, Norway; ^2^Faculty of Nursing, Sør-Trøndelag University College, Trondheim, Norway

## Abstract

The objective of this study was to increase knowledge of sexual abuse against older residents in nursing homes. A qualitative approach was used. Through a focus group interview with staff in nursing homes, the aim was to reveal employees' thoughts, experiences, and attitudes. Findings from the focus group interview show that sexual abuse of older residents is a taboo topic among health professionals. Acts of sexual abuse are difficult to imagine; it is hard to believe that it occurs. The fact that staff are not aware that it could happen, or have a hard time believing that it actually happens, can amplify the residents' vulnerable position as potential victims of abuse, and it makes it even more challenging to report or uncover such acts. The study highlights the need for education of all health care workers in Norway as well as more research on sexual abuse against older residents in nursing homes. Furthermore, there is a need for good policies and reporting systems, as an important step towards addressing sexual abuse of the aged in a more appropriate way. Further research must aim to reveal more about this taboo area.

## 1. Introduction

Abuse of older persons happens in all cultures and social classes, throughout the world. Assaults on older persons have long been a hidden phenomenon; it has been neglected in public and kept within the family. Sick and frail older persons are vulnerable because they often need help from family and paid carers, and this dependency increases the risk of abuse. Difficulties of detecting elder abuse can also be caused by the fact that many older persons have grown up in a culture where things should be kept secret within the family; thus it is not easy for older persons to tell anybody about the abuse [1].

Abuse of older persons is often defined as a repeated or single act, or lack of appropriate action, that happens in any relationship where there is an expectation of trust, which causes distress, injury, or suffering to an older person. Abuse is used as an umbrella term for threats, harassment, violence, and other acts violating a human being. Abuse can be of an emotional, physical, financial, or sexual nature [2].

In Norway we have no studies that with a certainty can quantify how many older persons are victims of violence and abuse, including sexual abuse. The oldest part of the population is either not covered by the various population surveys conducted, or they are reluctant to respond and are greatly underrepresented. Based on international research and clinical experience from Norway, it is estimated that four to six percent of older persons in Norway have been subjected to violence and abuse annually after the age of 65 [3].

Residents in long-term care settings are especially vulnerable to neglect, abuse, and exploitation, as they often are increasingly dependent on others and often suffer from dementia [4]. There is not yet an agreed definition of sexual abuse against older nursing home residents and in this study, the definition from the National Centre on Elder Abuse (NCEA), USA, is used: “Sexual abuse is defined as non-consensual sexual contact of any kind with an elderly person. Sexual contact with any person incapable of giving consent is also considered sexual abuse. It includes, but is not limited to, unwanted touching, all types of sexual assault or battery, such as rape, sodomy, coerced nudity, and sexually explicit photographing” [5].

According to Teaster and Roberto [4], sexual abuse is the last detected, perceived, and reported type of elder mistreatment. Because of the hidden nature of late-life sexual abuse and the difficulties in obtaining data on the topic, a paucity of research exists from domestic as well as institutional settings. A recently published literature review [6] revealed that only a few empirical studies have specifically addressed sexual abuse of older nursing home residents.

A study conducted by Burgess et al. [7] focused on sexual abuse of 20 nursing home residents reported to Burgess et al. as part of their forensic work. They found that the victims were predominately older, had cognitive deficits, and exhibited rape-related trauma symptoms. In 14 cases, the perpetrator was a nursing home staff member, and in three cases, the perpetrator was a resident. Eleven of the 20 victims died within 12 months of the assault, with half between the ages of 80 and 99. The lack of sensitivity of nursing home staff to the gravity of the assaults on the residents was the most striking observation.

Another study conducted by Teaster et al. [8] examined cases of male nursing home residents from 50 to 93 years who had been subjected to sexual abuse. More than 80% of the victims had decreased self-care with cognitive impairment and/or disability. Types of abuse were unwanted sexual attention, rape, and anal penetration. Of the suspected perpetrators 25% were other nursing home residents and 75% were staff.

Ramsey-Klawsnik et al. [9] focused on cases of 96 female and 27 male nursing home residents aged 60–101 years who had been sexually abused. 119 of the victims had reduced cognitive and physical functional level. Of the suspected perpetrators, the highest numbers were staff (*n* = 51) and other nursing home residents (*n* = 48). Only 32 of the cases had proof that abuse had taken place. No offenders were given legal punishment.

Ramsey-Klawsnik and Teaster [10] interviewed 46 staff members working in APS (Adult Protective Services) and other regulatory agencies in five states in the USA about reported sexual abuse of older persons in institutions. Interviewees had examined in total over 300 reported cases, and they indicated that they had lack of training in handling these types of cases. APS staff called for guidance, knowledge, and education in the area.

A study by Burgess et al. [11] examined the cases of 18 perpetrators who had committed sexual abuse of older residents in nursing homes. The perpetrators were from 16 to 83 years old and either staff (*n* = 15) or nursing home residents (*n* = 3). Two common denominators of the perpetrators were as follows: (1) they scored low on social competence; (2) they looked for victims who were frail and defenceless.

Internationally, there is little research on sexual abuse of older persons, and as far as we know, there is no research on this subject in Norway. Therefore, there is no record of how many older victims of sexual abuse there are in Norway, and the lack of knowledge about this taboo area is obvious [3]. The purpose of this study was to focus on sexual abuse of older residents in nursing homes. The study's specific aim was to explore nursing staff's experiences, thoughts, and attitudes about sexual abuse against older residents in nursing homes.

## 2. Method

Focus groups have become a widely accepted health research methodology [12]. The methodology effectively unearths the spectrum of participants' experiences and feelings and elicits further reflection on one's own perspective in the context of other convergent and divergent views in the group [13]. Although focus groups will not provide insight into the informants' individual life-world to the same extent as an in-depth interview, the conversation about similar experiences could contribute to important insights [14]. Collecting data through focus groups is considered a fruitful research method when aiming to illuminate experiences and attitudes. A research topic is explored through interaction between people purposively selected for their experiences with the research topic [13].

### 2.1. Participants and Procedure

In order to recruit staff with experience from nursing homes to reflect upon their own practice, the selection criteria for this study were women and men who had been permanently or temporarily employed for at least a year at a nursing home. Participants had to be registered nurses, licensed practical nurses, or nurse assistants.

Informants were recruited through a postgraduate course for health professionals. Six participants volunteered for the study. All six were female registered nurses, with an age range from mid-20s to late 30s. All had several years' experience from nursing homes. The participants worked at different nursing homes.

The interview was conducted in a meeting room at the campus. A few days before the interview, participants received a case story by e-mail (Case 1), describing a fictitious nursing home resident who was a victim of sexual assault. This served as an introduction to the topic. The interview guide consisted of open-ended questions related to the case, and the informants were encouraged to talk freely. The interview guide was intended as a checklist of key questions on the topic, which could be used to keep the thread of the interview.

Towards the end of the interview, a new case story (Case 2) was presented to the focus group. This case was based on a true story of a nursing home resident who had been raped by a male nurse. The purpose was to discover if the participants responded differently to this story. The interview lasted one hour and 45 minutes.

### 2.2. Data Analysis

The first author transcribed the tape recording. The purpose of transcription is, according to Krueger and Casey [13] and Malterud [15], to capture the conversation from interviews in a form that optimally presents what the interviewees intended to present. However, when a spoken conversation is being translated into text, it is inevitable that there will be some changes in the data, such as dialect expressions which will be transformed to ensure confidentiality.

Classic Analysis Strategy for focus groups, recommended by Krueger and Casey [13], was used as an analytical method for interpreting the data. Classic Analysis Strategy has been used as analysis in numerous projects. The method is to identify themes and categorize results. It is a systematic method that allows the analysis to be a visual and tangible process [13].

### 2.3. Ethical Issues

The Norwegian Social Science Data Services (reference number 32347) approved the research project. Prior to the study, participants were given thorough information via a letter which described the purpose of the study and a request to participate in the research project. A consent form was attached to the information letter. Informants were assured full anonymity, and information has been deidentified and is not recognizable in Section 3. It was emphasized that participating in the study was voluntary, and the informants were told that they had the opportunity to withdraw from the study at any time without explanation and with no consequences.

## 3. Findings

The purpose of this study was to explore thoughts, experiences, and attitudes about sexual abuse of nursing home residents from the perspective of nursing home staff. Findings from the focus group interview are presented below, using the main themes as headings.

### 3.1. * “This Does Not Happen … or Does It?”*

The immediate response from the participants to Case 1 was that it is unthinkable that someone can sexually assault a nursing home resident.* “Is it possible? I have never heard or experienced it,”* said one nurse. Another said:* “In such a situation it is not the first thing that would strike me … that it might be something like that [sexual abuse] in the picture … The case … It was such an eye-opener and this is probably happening more often than we realize.”* Most of the nurses had neither heard of nor experienced a patient who had been victim of sexual abuse in nursing homes.* “This does not happen … or does it?”* asked one participant. It appeared during the interview that two of the nurses had experienced incidents of sexual abuse of residents at their workplace. In these two cases, the perpetrators were, respectively, a relative and a friend. In one of the cases action in relation to the offence was undertaken; in the other nothing was done. The participants stated that they found it hard to realize that sexual abuse probably occurs in nursing homes and institutions for older persons. However, during the interview the nurses agreed that it would be naive of them to believe that such abuse did not happen.

### 3.2. *“It's Like a Horror Movie!”*

The topic seemed to raise strong reactions from the participants. All of them expressed negative feelings, and one nurse said:* “I am sad and angry because it is so hard … surely these things happen.” “I'm feeling sad”* said another. Anger, sadness, horror, feeling unwell, and nausea were emotions the participants expressed.* “It feels like a horror movie!”* said one nurse.* “It is taboo and difficult to talk about,”* said another. The participants reacted strongly to the fact that possible perpetrators often have free access to vulnerable older nursing home residents, and together with a huge lack of attention on the topic, this leads to lack of effort to protect and safeguard possible victims. One participant said:* “I feel sick, and I feel emotionally bad talking about such a topic”*.

### 3.3. *“Should Notify, but to Whom?”*

The focus group discussed what actions they would have taken if a resident had been victim of sexual assault. All participants agreed that they would have reported the incident. There was however considerable uncertainty in the group about whom it should be reported to. One participant put it this way:* “Yes, this must be reported to the police! I would have called the police right away.”* Some would call the police, others were unsure about whether their immediate leader should be notified first. All participants would investigate who the perpetrator was and take action. However, there was great uncertainty about the procedures and what documentation should be given and which agencies should be notified in such a case.* “You should notify someone, but whom?”* asked one participant. The nurses also stated that the duty of client confidentiality could be challenging,* “Am I allowed to not go ahead with it?”* asked one nurse. If the resident does not wish to proceed with the case or suffer from dementia, it will, according to the participants, offer a greater challenge. One participant said:* “I would have to talk to someone about it … but it's very difficult! I have a responsibility to stop it [the offence].”* Later in the discussion, the participants agreed that even where there is a matter of confidentiality such cases must be reported. First, to the department head, but such cases must be taken further and not be left in the document pile and “forgotten.” The participants agreed that they themselves should follow up the case, if they did not it could just “disappear” in the system. The nurses also agreed that sexual abuse, due to its nature, should not be part of the standard reporting form for deviations from quality but that there ought to be a separate reporting system for abuse cases. The participants expressed scepticism to what extent such matters would be taken seriously by the police.* “I think that such matters will soon be dropped by the police,”* said one of the nurses.

### 3.4. *“It's Not so Important with Older People …”*

The focus group discussed that negative attitudes towards older people might be one reason why a spotlight is not put on elder abuse, including sexual abuse. They questioned the fact that there are no requirements to report a criminal record when working with older nursing home residents, when it is required for work with other vulnerable groups such as children and persons with developmental disabilities. The participants believed that having to reveal a criminal record could be an important step in taking the older residents' safety seriously, as well as being an important part in the prevention of sexual abuse.* “When you are working with children and persons with developmentally [sic] disabilities you have to show a police certificate, so why should we not do it when we work with older nursing home residents?”* asked one nurse. Another said:* “I think the reason we do not need to submit proof of good conduct in nursing homes is due to staffing difficulties.”*

The participants were upset because there is no requirement for police clearance to work with older persons. One participant expressed it in the following way:* “I am thinking that it is due to ageism that one does not need to provide any police certification for working with older persons, it does not matter what happens to them, it is not so important with older people.”*

The focus group concluded that primarily it is the attitudes of those who work with the older nursing home residents that are important.* “If you are a perpetrator it does not matter whether you are a nurse or a nurse assistant … It is primarily the attitude!”* The participants discussed further what makes someone sexually assault a nursing home resident. If it is another resident who is the predator, it is usually a person who is demented or cognitively impaired. If the predator is a staff member, it is because this is about power and the person may be mentally ill. One participant said:* “I believe that when you sexually assault an older person, you have a disease in the same way as paedophilia.”* The participants were asked whether they had heard the term gerontophilia and the word's meaning was explained. None of them had heard the concept before. The discussions around gerontophilia led the participants to a discussion on older people and sexuality. It appeared that it is taboo to talk about seniors and sexuality. Therefore, it is difficult to imagine sexual abuse against older persons. One informant said:* “When you think of older people, sexuality is not part of the game, you are old and you are done with that.” *The participants agreed that there is little information about late-life sexuality. This is, according to the nurses, because older persons' sexual expressions are often hidden. It is therefore difficult to know how to deal with sexual acting-out behaviours in people with dementia in nursing homes. The informants called for education about sexuality and older persons.

### 3.5. *“There Is Need for Knowledge about Sexual Abuse against Older Persons”*

The informants suggested that knowledge about sexual abuse of older persons should be addressed in the education of health professionals. None of the participants were taught about the subject during their basic education.* “There is no focus on this in basic training. I call for knowledge about sexual abuse against older people in the education system,”* one informant said. The participants agreed that this topic should be included as part of the basic training of health professionals in order to create awareness of the issue. The informants believed that lack of education was caused by the anathema of the subject.* “You have to address this at many levels before the taboo is overcome, both educationally and in the community,”* said one informant. The focus group discussed the need for an information booklet about abuse of older persons in general, as well as specific information about sexual abuse. These booklets should be made available to educational institutions, as well as the practice field.* “This should be addressed at the national level,”* one informant said. The focus group also suggested use of ethical reflection groups in nursing homes.* “This is an uncomfortable topic, but I get engaged when we sit and talk about it … you get the focus on an important topic,”* one participant said.

### 3.6. *“You Become a Bit Paranoid—Going around and Suspecting Everyone”*

The discussions about sexual abuse against older persons in nursing homes led to a concern about ethics. Many of the participants were afraid that focusing on sexual abuse of older persons could make health personnel unsure during help with personal care. If residents do not want their private parts to be washed, would it be a violation to carry out the task? Another ethical challenge that raised discussion in the group was if a colleague was suspected of abuse. Some stated that it would be very difficult having to go around suspecting a colleague. One of the nurses put it like this:* “You are the little paranoid when … Going around and suspecting everyone.”* The participants viewed the issue of sexual abuse against older residents in nursing homes as a challenge, since the subject can easily lead to hysteria, in the sense that everyone goes around and suspects everyone else. They drew a parallel to when sexual abuse of children was put on the agenda. When it comes to reporting a possible perpetrator, the participants would notify the authorities even if the perpetrator was a well-liked colleague. They viewed it as a problem if a colleague suspected of abuse was fired from the nursing home and then was hired at another nursing home. The participants agreed that they would be willing to sacrifice their authorization to find out who the perpetrator was. “*If they want to take away my authorization because I have managed to find a perpetrator, yes, then they can do it,”* said one participant.

## 4. Discussion

Nursing home residents' dependency on others makes them vulnerable members of society [4]. Another important factor is that sexuality in older age is still taboo. The focus group participants said that because of this taboo, it might be hard to imagine that sexual abuse against older residents happens. The group agreed that a common stereotype perception is that sexuality and older people do not match. Older people are done with sexuality. The informants therefore asked for education about late-life sexuality. Without instruction and guidance on the subject, it is also difficult to know how to handle sexual acting-out behaviours in people with dementia. To understand why sexual abuse of older residents in nursing homes occurs and how to prevent this, it is important to raise an awareness of the issue in the community and among health professionals. Connolly et al. [16] say that by not having knowledge of sexuality among older persons and of sexual abuse against older persons, we are undermining their health, safety, and well-being.

The focus group discussed what kind of person would commit sexual abuse against vulnerable older nursing home residents. The participants concluded that if a coresident was the perpetrator of sexual assault, it usually was because this person had dementia or was cognitively impaired. If the abuser was a staff member, the reason could be an issue of power or because the person was mentally ill. Studies have proved both staff and coresidents to be perpetrators of sexual abuse [4, 8]. One nurse compared sexual abuse of older persons with paedophilia. None of the interviewees had heard of the word gerontophilia. According to Burgess et al. [17] gerontophiliacs is a group of assailants who often look for a job in nursing homes. The participants in the focus group expressed their surprise to the fact that there is a term that explains why some people are sexually attracted to older persons.

Requests for criminal records would be an important signal that the government and the nursing homes take the safety of older people seriously. According to Ramsey-Klawsnik et al. [9] it is of concern that nursing homes do not check employees' records. When a previously convicted offender has free access to older persons, the risk of further abuse is high.

Participants in the focus group did not believe that nursing home residents would easily be believed in abuse cases, especially if the resident had dementia. They believed that it would be more likely that the older person would be put on antidepressants. In addition, the nursing home resident would probably be suspected of incipient dementia or depression or having received too little sleep or stimuli rather than be believed. Studies have shown that older victims of sexual assault may experience an increase in mental and cognitive symptoms, such as withdrawal, stress, depression, and confusion [7, 18].

The fact that there is a need for knowledge about sexual abuse of residents in nursing homes was confirmed by the informants' strong emotional response to the subject in the interview. Connolly et al. [16] claim that lack of knowledge concerning sexual abuse of older persons is undermining their health, security, and welfare.

If employees are not aware of or have a hard time believing that this can happen, it can make it difficult to detect abuse. The interviewees pointed out that by discussing the topic they became more aware that this is something that occurs. Discussion on the theme contributed, according to the informants, to creating an awareness of their role and responsibility as healthcare professionals of having to attend to nursing homes residents' health and safety. The informants also called for lectures about sexual abuse against seniors as part of the education of health professionals. They believed that teaching on the subject should be included as part of the basic training of healthcare workers so that health professionals at an early stage could be aware of the subject. This view is supported by a study of 46 APS workers in the USA [10]. An important part of being able to address sexual abuse of older persons seriously is the development of teaching and tutoring programmes for health professionals. Information booklets, certificates of good conduct, and guidelines were mentioned as important. The participants also stressed the need for a separate reporting system for elder abuse in Norway, where healthcare professionals were obliged to report any case of elder abuse.

## 5. Methodological Considerations

The purpose of this study was to gain an insight into a still taboo and sensitive topic and therefore not easily researched. Protecting anonymity and ensuring informed consent are important principles in research ethics and should be of special concern when dealing with such a sensitive topic. The procedures for informing the participants and the signing of informed consent forms used in this study have been in accordance with these requirements.

The use of a focus group was considered an appropriate method for collecting data in this study, and the response from the participants confirmed this. At the end of the interview, the participants were asked whether they would feel freer to share experiences and thoughts if it was an individual interview. Two of the participants confirmed that they perhaps would have felt freer because in the focus group it was easy to be more bound by the others' opinions. However, the majority expressed that the challenging nature of the topic was easier to discuss in a group and that they felt more inspired by sharing their thoughts with the rest of the group.

The study has some limitations that have to be mentioned. The homogeneity of the group may have limited a more nuanced insight into the topic, since all six were female registered nurses, with an age range from mid-20s to late 30s, and all had worked for several years in nursing homes.

## 6. Concluding Remarks

The purpose of this study was to explore nursing staffs' thoughts, experiences, and attitudes about sexual abuse against older nursing home residents. Findings from the focus group show that sexual abuse of older persons is still taboo among health professionals. For many, sexual abuse of nursing home residents is so unthinkable that it is difficult to believe that it occurs. The fact that nursing staff are not aware that it could happen, or have a hard time believing that it actually happens, can amplify older persons' vulnerable position as potential victims of abuse. It makes it even more challenging to uncover abuse. Findings from the focus group show that there is a need for knowledge and further research on the topic. Furthermore, there is a need for good policies and reporting systems, as an important step in addressing sexual abuse of older persons seriously. Further research is needed to reveal more about this taboo area.
